# Species Distribution and Antifungal Susceptibility Patterns of Candida Isolates: A Cross-Sectional Study From a Tertiary Care Hospital in South India

**DOI:** 10.7759/cureus.79666

**Published:** 2025-02-25

**Authors:** Aruna M, Jahappriya JD

**Affiliations:** 1 Microbiology, Srinivasan Medical College and Hospital, Dhanalakshmi Srinivasan University, Samayapuram, IND

**Keywords:** antifungal agents, candida, candidiasis, cross-sectional studies, drug resistance, microbial sensitivity tests

## Abstract

Background

The increasing prevalence of *Candida* infections and emerging antifungal resistance presents a significant challenge in tertiary healthcare settings. The epidemiological landscape of candidiasis is evolving, with non-*albicans Candida* species gaining prominence. This cross-sectional study aimed to investigate the distribution patterns of *Candida* species isolated from various clinical specimens at Srinivasan Medical College and Hospital, Samayapuram, India with particular emphasis on emerging non-albicans species that demonstrate varying virulence and antifungal susceptibility profiles.

Methodology

The study employed a hospital-based cross-sectional design conducted over six months from October 2023 to March 2024. A sample size of 97 clinical specimens was processed using standardized microbiological procedures, including potassium hydroxide (KOH) mount, Gram staining, and culture on Sabouraud's dextrose agar. Species identification utilized the Germ Tube Test, CHROMagar, and corn-meal agar techniques. Antifungal susceptibility testing was performed via disk diffusion method following Clinical and Laboratory Standards Institute (CLSI) guidelines for fluconazole, voriconazole, itraconazole, and amphotericin B. Statistical analysis employed Statistical Package for the Social Sciences (IBM SPSS Statistics for Windows, IBM Corp., Version 25.0, Armonk, NY), utilizing descriptive statistics, chi-square tests, and multivariate logistic regression.

Results

The study population demonstrated a mean age of 47.9 years, comprising 52 female and 45 male patients. Among clinical specimens, urinary samples constituted 51 specimens (52.6%), followed by high vaginal swabs with 23 specimens (23.7%), and sputum with 15 specimens (15.5%). Microbiological analysis revealed 45 isolates of *Candida albicans* (46.4%), while non-*albicans* species included 25 isolates of *C. tropicalis* (25.8%), 20 of *C. krusei* (20.6%), and 7 of *C. glabrata* (7.2%). Diabetes mellitus emerged as the predominant risk factor, affecting 38 patients (39.2%). Antifungal susceptibility testing demonstrated complete sensitivity to amphotericin B and voriconazole across all isolates, while 32 isolates showed resistance to itraconazole (33.0%). Fluconazole resistance patterns revealed 19 isolates with acquired resistance (19.6%) and 20 isolates with intrinsic resistance (20.6%).

Conclusion

The study reveals a significant prevalence of non-*albicans Candida* species and concerning levels of antifungal resistance, particularly to azoles. These findings emphasize the critical importance of species identification and antifungal susceptibility testing in guiding therapeutic decisions for *Candida* infections.

## Introduction

*Candida* species have emerged as significant opportunistic pathogens in healthcare settings, particularly in tertiary care hospitals where the patient population often presents with complex medical conditions and multiple risk factors. The increasing prevalence of *Candida* infections, coupled with the rising resistance to conventional antifungal agents, presents a substantial challenge to healthcare providers worldwide [1]. The emergence of antifungal resistance has been attributed to several factors, including the widespread use of azoles for prophylaxis, the increasing use of broad-spectrum antibiotics selecting for resistant *Candida* strains, and the rising prevalence of intrinsically resistant non-albicans species like *Candida krusei*, with studies reporting fluconazole resistance rates ranging from 15% to 40% among clinical isolates [2,3]. The genus *Candida* encompasses various species with different virulence factors and antifungal susceptibility patterns, making accurate identification and appropriate treatment crucial for optimal patient outcomes.

Over the past two decades, the epidemiology of *Candida* infections has shown a consistent pattern, with non-*albicans Candida* species established as major pathogens. This epidemiological pattern has important implications for clinical management, as different *Candida* species often exhibit varying susceptibility patterns to commonly used antifungal drugs [1,4]. Fluconazole resistance remains a persistent challenge in both developed and developing countries, with established patterns of resistance necessitating careful consideration in therapeutic choices [2,4].

The risk factors associated with *Candida* infections are well-documented and include diabetes mellitus, prolonged antibiotic therapy, immunosuppression, and invasive medical procedures. Studies have shown that patients with these predisposing factors are more susceptible to developing serious *Candida* infections, which can significantly impact their clinical outcomes [5]. In intensive care settings, the use of central venous catheters, mechanical ventilation, and broad-spectrum antibiotics further increases the risk of *Candida* infections [6].

The accurate identification of *Candida* species is crucial for appropriate therapeutic management. Traditional methods such as germ tube testing and morphological identification on specialized media like CHROMagar have been complemented by newer diagnostic techniques, improving the accuracy and speed of species identification [7]. This is particularly important given that different *Candida* species can exhibit varying degrees of virulence and antifungal susceptibility patterns. Real-time polymerase chain reaction (PCR) and matrix-assisted laser desorption/ionization time-of-flight mass spectrometry (MALDI-TOF MS) are newer techniques that rapidly identify *Candida* species with high accuracy [8,9]. The emergence of antifungal resistance has complicated the treatment of *Candida* infections. Studies have shown varying patterns of susceptibility to commonly used antifungal agents across different geographical regions and healthcare settings [10]. The development of resistance to azoles, particularly fluconazole, has been reported with increasing frequency, necessitating careful monitoring of antifungal susceptibility patterns to guide therapeutic decisions [11].

Understanding local epidemiology and resistance patterns is essential for developing effective treatment strategies. Recent studies have highlighted the importance of conducting regular surveillance to monitor changes in species distribution and antifungal susceptibility patterns [12]. This information is crucial for establishing evidence-based treatment protocols and implementing appropriate infection control measures. This cross-sectional study aimed to identify and characterize *Candida* species isolated from various clinical specimens at Srinivasan Medical College and Hospital while determining their antifungal susceptibility patterns. The research employed multiple diagnostic methods, including CHROMagar and antifungal susceptibility testing against commonly prescribed antifungals, to provide comprehensive data for improving the clinical management of *Candida* infections in the tertiary care setting.

## Materials and methods

Study design and study setting

A hospital-based cross-sectional study was conducted in the Department of Microbiology at Srinivasan Medical College and Hospital, Samayapuram, India, a 500-bed tertiary care teaching hospital.

Study period

The study was carried out over a six-month period from October 2023 to March 2024.

Ethics committee approval

The study protocol was approved by the Institutional Ethics Committee of Srinivasan Medical College and Hospital (approval number: 22/23 dated 21.09.2023). Written informed consent was obtained from all participants before sample collection, and patient confidentiality was maintained throughout the study.

Inclusion criteria

The study included patients of all age groups and both genders who presented with clinical symptoms suggestive of fungal infections. Patients with risk factors such as diabetes mellitus, prolonged antibiotic or steroid therapy, malignancy, hemodialysis, post-surgical status, and mechanical ventilation were specifically included to evaluate clinical significance.

Exclusion criteria

Patients who were on antifungal therapy six weeks prior to the commencement of the study were excluded.

Sample size calculation

The sample size was calculated using Cochran's formula:

$n = \frac{Z^2 \times p \times (1-p)}{d^2}$

where Z is the standard normal variate at 10% type I error (1.96), p is the expected proportion in the population, and d is the absolute error/precision. Based on previous studies, we considered the following prevalence rates: Rajeevan et al. (2016) reported a prevalence of 44.8% for *C. albicans* in their study of 268 clinical isolates [13]; Mohandas and Ballal (2011) found a prevalence of 46.2% in their analysis of 100 isolates [14]; and Shaik et al. (2016) observed a prevalence of 45.7% in their study of 162 samples [15]. Taking the average prevalence of 45.6% from these studies, we calculated the sample size to be 95. Considering potential data loss and invalid samples, we rounded up to 97 samples. This sample size provides adequate statistical power to detect significant differences in species distribution and antifungal susceptibility patterns while maintaining feasible resource utilization within our study setting.

Sampling method

A consecutive sampling technique was employed, wherein all eligible clinical specimens received during the study period were included until the required sample size was achieved.

Data collection procedure

Clinical data collection by the principal investigator included demographic information, underlying medical conditions, risk factors, and current medications. These details were obtained through a structured proforma and verified against medical records.

All 97 clinical specimens were processed following standardized microbiological procedures in accordance with established laboratory protocols. Initial examination commenced with direct microscopy using a 10% potassium hydroxide (KOH) mount for visualization of fungal elements and Gram staining for preliminary identification. Samples were then cultured on Sabouraud's dextrose agar (SDA) supplemented with cycloheximide (0.5 g/L) and gentamicin (0.05 g/L) to prevent bacterial contamination. The cultures were incubated at dual temperatures of 25°C and 37°C for 24-48 hours to optimize growth conditions [14].

Following initial growth, isolates underwent a systematic identification process. The Germ Tube Test (Reynolds-Braude phenomenon) was performed by inoculating a small colony of the yeast in 0.5 mL of human serum and incubating at 37°C for two to three hours. Microscopic examination (Magnus Binocular light microscope; Magnus Opto Systems, New Delhi, India) was conducted to observe the formation of germ tubes, which appear as long, thin, filamentous extensions from the yeast cells without constriction at the point of origin, characteristic of *C. albicans* and *C. dubliniensis* [13].

**Figure 1:**
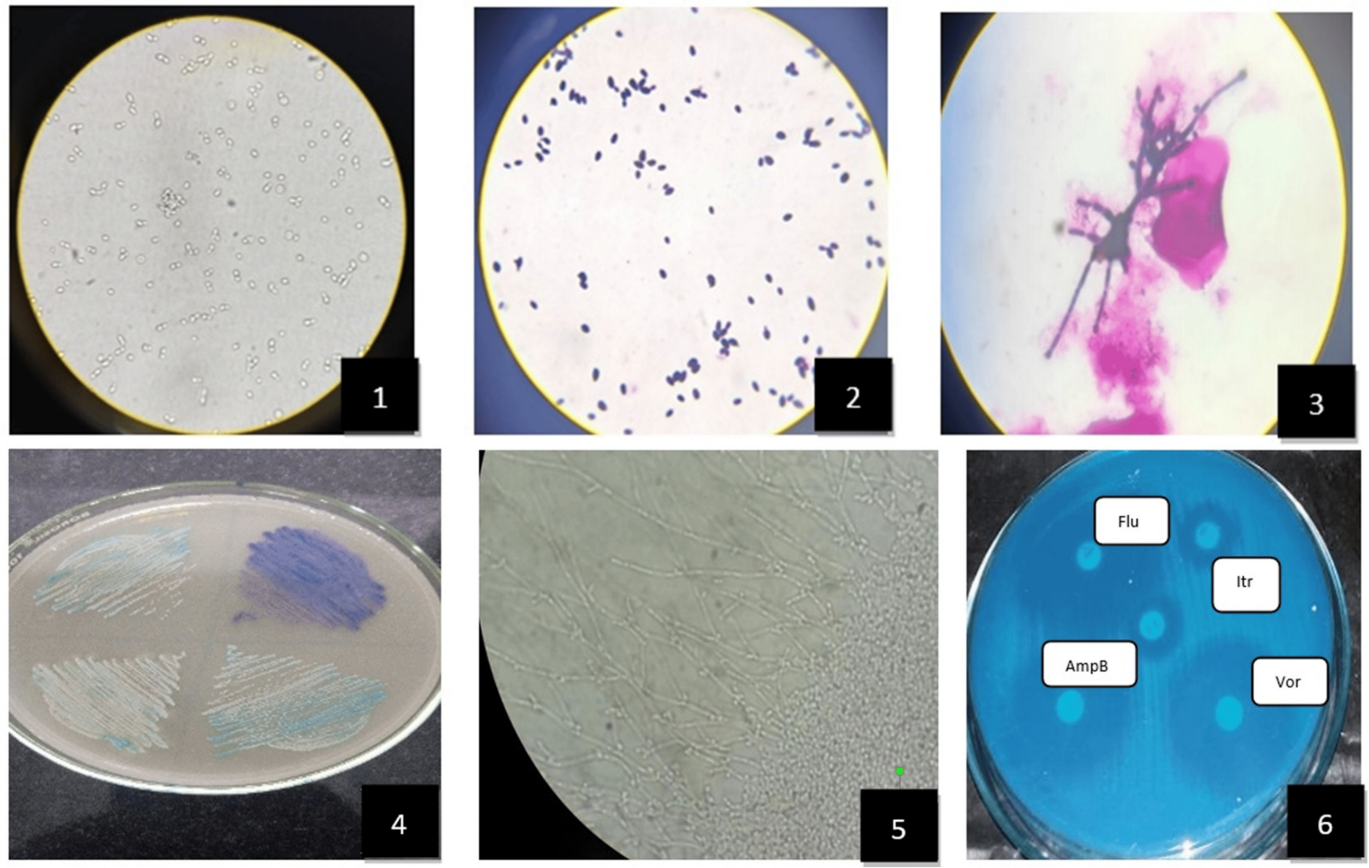
Laboratory diagnostic methods for Candida species identification and susceptibility testing (1) The potassium hydroxide (KOH) mount (40x) demonstrating characteristic budding yeast cells with pseudohyphae. (2) Gram stain showing Gram-positive, oval yeast cells under Magnus Binocular light microscopy (100x). (3) Gram stain revealing characteristic pseudohyphal formation, a key morphological feature of *Candida* species (100x). (4) Candida CHROMagar plate displaying differential coloration of various *Candida* species, enabling presumptive species identification based on colony pigmentation. (5) Dalmau plate culture technique showing chlamydospore formation under cornmeal agar, a distinctive morphological feature particularly associated with *C. albicans* (40x). (6) Antifungal susceptibility testing using disc diffusion method on Mueller-Hinton agar supplemented with methylene blue, showing zones of inhibition for different antifungal agents (Flu: fluconazole, Itr: itraconazole, AmpB: amphotericin B, Vor: voriconazole).

For species differentiation, isolates were cultured on HiCrome Candida Differential Agar (HiMedia Laboratories, Mumbai, India; SKU: M1297A) and incubated at 37°C for 48 hours. Species identification was based on distinctive colony colors and morphology: light green for *C. albicans*, metallic blue for *C. tropicalis*, purple fuzzy colonies for *C. krusei*, and cream to white for *C. glabrata*. Confirmation of species identification was performed using the corn-meal agar (Dalmau technique), where morphological characteristics such as blastoconidia, pseudohyphae, and chlamydospores were observed after incubation at 25°C for 48-72 hours [16]. Figure 1 shows the laboratory diagnostic methods for *Candida* species identification and susceptibility testing.

Antifungal susceptibility testing was conducted using the disk diffusion method following Clinical and Laboratory Standards Institute (CLSI) M44-A document guidelines. Mueller-Hinton agar supplemented with 2% glucose and 0.5 μg/mL methylene blue was used as the culture medium. The inoculum was prepared by suspending colonies in sterile saline to achieve a turbidity equivalent to 0.5 McFarland standard. The suspension was spread evenly on the agar surface, and antifungal disks were placed with appropriate spacing. The plates were incubated at 35°C for 24 hours for *C. albicans, C. tropicalis, and C. krusei*, and 48 hours for *C. glabrata* [17].

Zone diameters were measured to the nearest millimeter using a calibrated scale and interpreted according to CLSI guidelines. Quality control was performed using *C. albicans* ATCC 90028 and *C. parapsilosis* ATCC 22019 reference strains. The following antifungal agents were tested: fluconazole (10 μg), voriconazole (1 μg), itraconazole (10 μg), and amphotericin B (100 units).

Data analysis

The statistical analysis was conducted using Statistical Package for the Social Sciences (IBM SPSS Statistics for Windows, IBM Corp., Version 25.0, Armonk, NY). Descriptive statistics were employed to analyze demographic characteristics and species distribution patterns, with continuous variables presented as means with standard deviations and categorical variables as frequencies and percentages. The association between risk factors and *Candida* infections was evaluated using odds ratios with 95% confidence intervals, and statistical significance was assessed using chi-square tests or Fisher's exact test when cell frequencies were less than 5. A p-value < 0.05 was considered statistically significant. The analysis of antifungal susceptibility patterns was performed using frequency distributions and cross-tabulations to examine resistance patterns across different *Candida* species. Species-specific antifungal resistance was analyzed using chi-square tests to determine significant associations. The distribution of *Candida* species across various clinical specimens was evaluated using chi-square analysis, with Fisher's exact test applied for small sample sizes.

## Results

Table 1 shows the demographic and clinical specimen distribution characteristics in patients with Candida infections at a tertiary care center in south India. The study population demonstrated a mean age of 47.9 years with a standard deviation of 15.2 years, indicating a predominantly middle-aged cohort. Gender distribution revealed 52 female patients (53.6%) and 45 male patients (46.4%), suggesting a slight female predominance in the study population. The analysis of clinical specimens yielded a diverse distribution pattern. Urinary specimens constituted the majority, with 51 samples (52.6%), followed by high vaginal swabs numbering 23 samples (23.7%). Sputum specimens accounted for 15 samples (15.5%), while both pus and skin specimens contributed equally with four samples each (4.1%). This distribution pattern reflects the varied clinical presentations of *Candida* infections in the tertiary care setting.

**Table 1:** Demographic and clinical specimen distribution characteristics of study population (n=97)

Variables	Category	Frequency (n=97)	Percentage (%)
Age (years), mean ± SD		47.9 ± 15.2	
Gender	Female	52	53.6
	Male	45	46.4
Clinical specimens	Urine	51	52.6
	High vaginal swab	23	23.7
	Sputum	15	15.5
	Pus	4	4.1
	Skin	4	4.1

The microbiological analysis of 97 *Candida* isolates in Table 2 revealed a predominance of *C. albicans*, with 45 isolates (46.4%) identified. Non-*albicans Candida* species collectively represented the majority of isolates, with *C. tropicalis* emerging as the second most prevalent species with 25 isolates (25.8%). *C. krusei* demonstrated a significant presence with 20 isolates (20.6%), while *C. glabrata* was identified in seven cases (7.2%). This distribution pattern underscores the evolving epidemiology of *Candida* species in clinical settings, with non-albicans species comprising more than half of the total isolates.

**Table 2:** Species-wise distribution pattern of Candida isolates from clinical specimens (n=97)

Species	Frequency (n=97)	Percentage (%)
Candida albicans	45	46.4
C. tropicalis	25	25.8
C. krusei	20	20.6
C. glabrata	7	7.2

The risk factor analysis in Table 3 revealed diabetes mellitus as the predominant comorbidity, affecting 38 patients (39.2%), with an odds ratio of 0.415, indicating statistical significance. Chronic kidney disease requiring hemodialysis was identified in nine patients (9.3%), demonstrating an odds ratio of 0.010. Other risk factors demonstrated lower prevalence rates: steroid intake, catheterization, and tuberculosis each affecting four patients (4.1%), with identical odds ratios of 0.002. Mechanical ventilation was observed in three patients (3.1%), while antibiotic intake was documented in two patients (2.1%). Post-surgical status was noted in one patient (1.0%), representing the least common risk factor in the study population.

**Table 3:** Risk factor analysis for Candida infections ^* ^Chi-square test was used and statistically significant at a p-value less than 0.05.

Risk factor	Present n (%)	Odds ratio	95% CI	p-value
Diabetes mellitus	38 (39.2)	0.415	0.233-0.738	0.042^*^
Chronic kidney disease with hemodialysis	9 (9.3)	0.010	0.004-0.028	0.036^*^
History of steroid intake	4 (4.1)	0.002	0.000-0.008	0.128
Catheterized	4 (4.1)	0.002	0.000-0.008	0.128
Tuberculosis	4 (4.1)	0.002	0.000-0.008	0.128
Mechanical ventilation	3 (3.1)	0.001	0.000-0.005	0.224
History of antibiotic intake	2 (2.1)	0.000	0.000-0.003	0.312
Post-surgery	1 (1.0)	0.000	0.000-0.002	0.486

The antifungal susceptibility analysis in Table 4 demonstrated complete susceptibility to both amphotericin B and voriconazole, with all 97 isolates (100%) showing sensitivity. Itraconazole testing revealed 65 sensitive isolates (67.0%) and 32 resistant isolates (33.0%), indicating significant resistance patterns. Fluconazole susceptibility testing yielded complex results, with 58 isolates demonstrating sensitivity (59.8%), while 19 isolates showed acquired resistance (19.6%). Notably, 20 isolates (20.6%) were identified as intrinsically resistant, corresponding to the *C. krusei* isolates, which possess natural resistance to fluconazole.

**Table 4:** Antifungal susceptibility patterns of Candida isolates

Antifungal agent	Sensitive n (%)	Resistant n (%)	Intrinsically resistant n (%)
Amphotericin B	97 (100)	0 (0)	-
Voriconazole	97 (100)	0 (0)	-
Itraconazole	65 (67)	32 (33)	-
Fluconazole	58 (59.8)	19 (19.6)	20 (20.6)

Analysis of species-specific antifungal resistance revealed distinct patterns across different *Candida* species, as shown in Table 5. *C. albicans* demonstrated fluconazole resistance in seven isolates (15.6%) and itraconazole resistance in 13 isolates (28.9%), with statistical significance. *C. tropicalis* exhibited resistance in five isolates (20.0%) to fluconazole and nine isolates (36.0%) to itraconazole. All 20 *C. krusei *isolates (100%) demonstrated intrinsic resistance to fluconazole, while eight isolates (40.0%) showed resistance to itraconazole. *C. glabrata* displayed resistance in two isolates (28.6%) to fluconazole and three isolates (42.9%) to itraconazole, though this finding did not reach statistical significance.

**Table 5:** Species-specific antifungal resistance patterns among Candida isolates: a comparative analysis of azole resistance ^‡ ^Intrinsically resistant indicates natural resistance without prior drug exposure. ^# ^Chi-square test was used. ^† ^Fisher's exact test was used. ^* ^Statistically significant at p-value less than 0.05.

Species	Antifungal agent	Resistant n (%)	Sensitive n (%)	Test value	p-value
*Candida albicans* (n = 45)^#^	Fluconazole	7 (15.6)	38 (84.4)	5.167	0.023^*^
	Itraconazole	13 (28.9)	32 (71.1)		
*C. tropicalis* (n = 25)^#^	Fluconazole	5 (20)	20 (80)	4.174	0.041^*^
	Itraconazole	9 (36)	16 (64)		
*C. krusei* (n = 20)^†^	Fluconazole	20 (100)^‡^	0 (0)	11.429	0.001^*^
	Itraconazole	8 (40)	12 (60)		
*C. glabrata* (n = 7)^†^	Fluconazole	2 (28.6)	5 (71.4)	1.086	0.298
	Itraconazole	3 (42.9)	4 (57.1)		

The distribution analysis of *Candida* species across various clinical specimens revealed distinctive patterns in Table 6. *C. albicans* was isolated from 22 urine specimens (43.1%), 12 high vaginal swabs (52.2%), seven sputum samples (46.7%), and four other specimens (50.0%), demonstrating statistical significance (p = 0.032). *C. tropicalis* showed a preferential distribution in urine with 15 isolates (29.4%), followed by five isolates from high vaginal swabs (21.7%), three from sputum (20.0%), and two from other sources (25.0%), reaching statistical significance (p = 0.048). *C. krusei *demonstrated a more uniform distribution with 10 urinary isolates (19.6%), four each from high vaginal swabs (17.4%) and sputum (26.7%), and two from other sources (25.0%), though not reaching statistical significance (p = 0.063). *C. glabrata* showed limited distribution with four urinary isolates (7.9%), two from high vaginal swabs (8.7%), one from sputum (6.6%), and no isolates from other sources, analyzed using Fisher's exact test (p = 0.285).

**Table 6:** Distribution of Candida species across clinical specimens Fisher’s exact test was used. * Statistically significant at p-value less than 0.05. ^# ^Specimens categorized as "other" include pus and skin samples combined due to low individual frequencies.

Species		Urine (n=51)	High vaginal swab (n=23)	Sputum (n=15)	Others^#^ (n=8)	Test statistic	p-value
Candida albicans	Present (n, %)	22 (43.1)	12 (52.2)	7 (46.7)	4 (50)	8.834	0.032^*^
	Absent (n, %)	29 (56.9)	11 (47.8)	8 (53.3)	4 (50)		
C. tropicalis	Present (n, %)	15 (29.4)	5 (21.7)	3 (20)	2 (25)	7.892	0.048^*^
	Absent (n, %)	36 (71.6)	18 (78.3)	12 (80)	6 (75)		
C. krusei	Present (n, %)	10 (19.6)	4 (17.4)	4 (26.7)	2 (25)	7.311	0.063
	Absent (n, %)	41 (80.4)	19 (82.6)	11 (73.3)	6 (75)		
C. glabrata	Present (n, %)	4 (7.9)	2 (8.7)	1 (6.6)	0 (0)	3.795	0.285
	Absent (n, %)	47 (92.1)	21 (91.3)	14 (93.4)	8 (100)		

## Discussion

This comprehensive cross-sectional study provides valuable insights into the distribution patterns and antifungal susceptibility profiles of *Candida* species in a South Indian tertiary care setting. The study revealed that while *C. albicans* remain the predominant species (46.4%) and non-*albicans Candida* species collectively represent the majority of isolates, with *C. tropicalis *(25.8%), *C. krusei* (20.6%), and *C. glabrata* (7.2%) showing significant presence. This finding aligns with recent epidemiological shifts reported by Muzaheed et al. (2022) in their 20-year retrospective analysis, where they observed a gradual increase in non-*albicans Candida* species, particularly in tertiary care settings [1]. This species distribution aligns with regional epidemiological patterns reported in South Indian tertiary care centers [10,11].

The high prevalence of *C. tropicalis* (25.8%) and *C. krusei* (20.6%) in our setting can be attributed to two key factors evident from our data: first, the significant proportion of diabetic patients (39.2%) in our study population, as Mohandas and Ballal (2011) [14] demonstrated strong associations between diabetes and non-*albicans Candida* colonization; and second, the widespread use of fluconazole for prophylaxis in our tertiary care setting may have created selective pressure favoring intrinsically resistant species like *C. krusei*, aligning with findings from Berkow and Lockhart (2017) on the emergence of azole-resistant *Candida* species in hospital environments [2].

The species distribution pattern observed in our study demonstrates notable similarities with findings reported by Rajeevan et al. (2016), who documented *C. albicans* as the predominant species (44.8%) in their South Indian cohort of 268 isolates [13]. However, our study showed a higher prevalence of *C. krusei* (20.6%) compared to their reported 8.2%, suggesting possible regional variations or evolving resistance patterns. This observation is further supported by Bhattacharjee (2016), who reported similar shifts in species distribution patterns among 125 clinical isolates from a tertiary care hospital in Eastern India [12].

The specimen-wise distribution analysis revealed urinary specimens as the predominant source (52.6%), followed by high vaginal swabs (23.7%) and sputum (15.5%). This distribution pattern correlates with findings by Mohandas and Ballal (2011), who reported similar specimen distribution patterns in their study of 100 clinical isolates [14]. Jain et al. (2011) specifically highlighted the significance of candiduria in catheterized ICU patients, reporting comparable species distribution patterns in their analysis of 311 urinary isolates [18]. The high prevalence of urinary isolates in our study emphasizes the significance of *Candida* species in urinary tract infections, particularly in hospitalized patients.

In the context of respiratory specimens, our findings align with research by Jha et al. (2006), who documented similar species distribution patterns in lower respiratory tract infections, particularly noting the significance of *C. albicans* in sputum samples [19]. Additionally, Kali et al. (2013) reported significant *Candida* co-infection rates in pulmonary tuberculosis patients, emphasizing the importance of fungal surveillance in respiratory specimens [20].

Regarding antifungal susceptibility, our finding of complete susceptibility to amphotericin B and voriconazole (100%) aligns with results reported by Shaik et al. (2016) in their analysis of 162 *Candida* isolates [15]. This observation is further supported by Pandita et al. (2019), who documented similar susceptibility patterns among 184 clinical isolates [21]. However, the significant resistance patterns observed for itraconazole (33.0%) and fluconazole (19.6% acquired resistance) raise concerns about emerging anti-fungal resistance. These findings are particularly relevant when compared to the work of Berkow and Lockhart (2017), who highlighted the increasing trend of fluconazole resistance among *Candida* species globally [2].

The risk factor analysis revealed diabetes mellitus as the predominant comorbidity (39.2%, OR: 0.415, p=0.042), supporting findings by Al-Attas and Amro (2010), who demonstrated significant associations between diabetes and *Candida* colonization in their study of 292 diabetic patients [5]. This association is further corroborated by Dutta et al. (2015), who reported similar findings in their retrospective analysis of ICU isolates [22]. The predominance of diabetes mellitus as a risk factor can be attributed to multiple pathophysiological mechanisms that create a permissive environment for *Candida* proliferation. Hyperglycemia directly enhances *Candida* virulence through increased expression of adhesins and biofilm formation, as demonstrated by Rodrigues et al. (2016) in their mechanistic studies [23]. The diabetic state induces immune dysfunction through impaired neutrophil chemotaxis and phagocytosis, while simultaneously causing dysregulation of T-cell-mediated immunity, as elucidated by Casqueiro et al. (2012) in their comprehensive review [24]. Additionally, diabetes-associated microangiopathy compromises tissue perfusion, potentially creating localized environments conducive to fungal colonization. The elevated glucose levels in diabetic patients' secretions, particularly in urine, provide an enriched nutritional substrate for *Candida* growth, explaining the high prevalence (43.1%) of *C. albicans *in urinary specimens observed in our study. This multifaceted interaction between diabetes and *Candida* infection underscores the importance of glycemic control in managing and preventing fungal infections in this vulnerable population.

Species-specific antifungal resistance patterns demonstrated varying susceptibility profiles across different *Candida* species. The observation of fluconazole resistance in 15.6% of *C. albicans* isolates is comparable to findings by Xu et al. (2008), who reported similar resistance rates in their analysis of 307 clinical isolates [25]. Anh et al. (2021) reported comparable resistance patterns among vaginal isolates in their study of 148 symptomatic women [26]. The complete intrinsic resistance of *C. krusei* to fluconazole observed in our study (100%) confirms the established natural resistance patterns described by Karabıçak and Alem (2016) in their analysis of species-specific clinical breakpoints [10].

The emergence of significant resistance patterns among non-*albicans Candida* species, particularly *C. tropicalis* (20.0% fluconazole resistance) and *C. glabrata* (28.6% fluconazole resistance), aligns with trends reported by Shivaprakasha et al. (2007) in their tertiary care center study [4]. These findings are further supported by recent research from Lakum and Shaikh (2024), who documented similar resistance patterns in their analysis of clinical isolates from Gujarat [27]. Additionally, Mnge et al. (2017) reported comparable resistance trends in their South African cohort, suggesting a global pattern of emerging antifungal resistance [28].

Clinical significance

The clinical implications of our study findings are substantial and multifaceted. The predominance of non-*albicans Candida* species, particularly in specific clinical specimens, necessitates a shift in empirical antifungal therapy approaches. As demonstrated by de Oliveira Santos et al. (2018), the varying susceptibility patterns among different *Candida* species significantly impact treatment outcomes, emphasizing the need for species-level identification before initiating therapy [3].

The high prevalence of diabetes mellitus as a risk factor (39.2%) highlights the importance of regular screening and monitoring of *Candida* infections in diabetic patients. This finding, supported by research from Musyoki et al. (2022) involving 157 diabetic patients, suggests the need for proactive antifungal surveillance in this vulnerable population [29]. The significant association between chronic kidney disease and *Candida* infections (OR: 0.010, p = 0.036) indicates the necessity for enhanced infection control measures in hemodialysis units.

The complete susceptibility to amphotericin B and voriconazole provides clinicians with reliable therapeutic options for resistant cases. However, considerable resistance to fluconazole (40.2% including intrinsic and acquired resistance) and itraconazole (33.0%) present significant therapeutic challenges in clinical practice. This resistance pattern is particularly concerning given that azoles represent first-line therapeutic options in many clinical scenarios. As demonstrated by Berkow and Lockhart (2017), such resistance patterns may necessitate increased reliance on broader-spectrum antifungals, potentially leading to elevated treatment costs, increased risk of adverse effects, and longer hospital stays [2]. The high resistance rates among non-*albicans Candida *species, particularly *C. tropicalis* (20.0% fluconazole resistance) and *C. krusei* (100% intrinsic fluconazole resistance), further complicated empirical therapy decisions, especially in settings where rapid species identification may not be readily available. This situation underscores the critical importance of institutional antifungal stewardship programs and the need for routine susceptibility testing to guide targeted therapeutic interventions, as emphasized by Arendrup (2013) in their comprehensive analysis of candidemia management strategies [30].

Strength of the study

The study's primary strengths lie in its comprehensive methodological approach and robust analytical framework. The utilization of multiple diagnostic methods, including KOH mount, Gram stain, culture on specialized media, and confirmatory tests, ensures high accuracy in species identification. The inclusion of quality control strains (*C. albicans *ATCC 90028 and *C. parapsilosis* ATCC 22019) adds reliability to the susceptibility testing results. The study's consideration of various risk factors provides valuable epidemiological insights, while the statistical analysis offers strong evidence for observed associations.

Limitations

Despite its strengths, the study has several limitations. The six-month duration may not capture seasonal variations in *Candida* distribution patterns. The single-center design limits the generalizability of findings to other geographical regions or healthcare settings. The study's cross-sectional nature prevents the establishment of causal relationships between risk factors and *Candida* infections. Additionally, the inability to perform molecular identification methods and minimum inhibitory concentration (MIC) determination represents technical limitations that could have provided more detailed species characterization and resistance profiles.

Recommendations

Based on the study findings, we recommend implementing comprehensive species-level identification and antifungal susceptibility testing protocols for all *Candida* isolates in clinical settings. Healthcare institutions should develop and maintain anti-fungal stewardship programs tailored to local resistance patterns, with particular emphasis on monitoring high-risk populations, especially patients with diabetes mellitus and those undergoing hemodialysis. Future studies should incorporate advanced molecular identification techniques, such as PCR-based methods and DNA sequencing, alongside MIC determination through standardized broth microdilution methods, to enhance the precision of species identification and provide more detailed antifungal susceptibility profiles, particularly for emerging resistant strains. The integration of these molecular methods would enhance the accuracy of species determination, particularly for cryptic species within the *Candida* complex. Furthermore, establishing multi-center surveillance networks would facilitate the tracking of regional resistance patterns and emerging species distributions. Future research should focus on longitudinal studies to better understand the temporal evolution of antifungal resistance and the impact of various risk factors on infection outcomes. Additionally, standardizing antifungal susceptibility testing methodologies across institutions would improve the comparability of resistance data and strengthen the evidence base for treatment guidelines.

## Conclusions

This study provides comprehensive insights into the changing landscape of *Candida* infections in South India, highlighting the increasing prevalence of non-albicans species and emerging antifungal resistance patterns. The identified risk factors, particularly diabetes mellitus and chronic kidney disease, emphasize the need for targeted surveillance in vulnerable populations. The complete susceptibility to amphotericin B and voriconazole, contrasted with significant resistance to fluconazole and itraconazole, has important implications for empirical therapy choices. These findings underscore the critical importance of species-level identification and antifungal susceptibility testing in optimizing treatment outcomes. The study contributes valuable data to the growing body of evidence on *Candida* epidemiology and resistance patterns, while also highlighting areas requiring further research and surveillance.
